# Synergistic Effect of Low Molecular Weight Polyethylenimine and Polyethylene Glycol Components in Dynamic Nonviral Vector Structure, Toxicity, and Transfection Efficiency

**DOI:** 10.3390/molecules24081460

**Published:** 2019-04-12

**Authors:** Bogdan Florin Craciun, Gabriela Gavril, Dragos Peptanariu, Laura Elena Ursu, Lilia Clima, Mariana Pinteala

**Affiliations:** “Petru Poni” Institute of Macromolecular Chemistry, Romanian Academy, Centre of Advanced Research in Bionanoconjugates and Biopolymers, Grigore Ghica Voda Alley, 41 A, 700487 Iasi, Romania; craciun.bogdan@icmpp.ro (B.F.C.); pricope.gabriela@icmpp.ro (G.G.); biodragos@gmail.com or peptanariu.dragos@icmpp.ro (D.P.); ursu.laura@icmpp.ro (L.E.U.)

**Keywords:** drug delivery, dynamic combinatorial chemistry, supramolecular assembly, squalene, DNA condensation

## Abstract

When studying polyethylenimine derivatives as nonviral vectors for gene delivery, among the important issues to be addressed are high toxicity, low transfection efficiency, and nucleic acid polyplex condensation. The molecular weight of polyethylenimine, PEGylation, biocompatibility and, also, supramolecular structure of potential carrier can all influence the nucleic acid condensation behavior, polyplex size, and transfection efficiency. The main challenge in building an efficient carrier is to find a correlation between the constituent components, as well as the synergy between them, to transport and to release, in a specific manner, different molecules of interest. In the present study, we investigated the synergy between components in dynamic combinatorial frameworks formed by connecting PEGylated squalene, poly-(ethyleneglycol)-bis(3-aminopropyl) and low molecular weight polyethylenimine components to 1,3,5-benzenetrialdehyde, via reversible imine bond, applying a dynamic combinatorial chemistry approach. We report comparative structural and morphological data, DNA binding affinity, toxicity and transfection efficiency concerning the ratio of polyethylenimine and presence or absence of poly-(ethyleneglycol)-bis(3-aminopropyl) in composition of dynamic combinatorial frameworks. In vitro biological assessments have revealed the fact that nonviral vectors containing poly-(ethyleneglycol)-bis(3-aminopropyl) and the lowest amount of polyethylenimine have significant transfection efficiency at N/P 50 ratio and display insignificant cytotoxicity on the HeLa cell line.

## 1. Introduction

Gene delivery holds great promise for correcting genetic defects and treating myriad of genetic and acquired diseases [1,2,3,4]. However, major problems in gene therapy are related to the development of efficient and targeted DNA carrier able to deliver large quantities of genetic material. Based on their design and properties, viral vectors currents show the best results in effective gene delivery [1,2,3,4,5,6]. Alternatively, all other approaches are based on nonviral gene delivery systems, which try to mimic the efficiency of viral vectors by artificial means [7,8,9,10]. Among the nonviral systems, the most explored building blocks are represented by cationic compounds, essentially polymers, which have shown significant development in the delivery of nucleic acids in the last decades [1,7,11,12]. Cationic polymers, represented commonly by polyethylenimine (PEI), including branched (B-PEI) and linear polyethylenimine (L-PEI), have become a safer alternative to viral vectors and have shown good transfection efficiency (TE) in different types of cells and their “proton sponge” characteristic [7,8,9,11,13,14]. PEI has the ability to bind DNA by electrostatic interaction to form small complexes (polyplexes) that are internalized into cells by endocytosis and can be localized to the nucleus as distinct structures [11,12,15]. It is commonly believed that the molecular weight of PEI for the most effective gene transfer ranges between 5 and 25 kDa [10,16]. Higher molecular weights lead to increased cytotoxicity, while low molecular weight PEI, on the other hand, has demonstrated a low toxicity in cell culture studies [7,17,18]. L-PEI contains only secondary amino groups, thus inducing reduced cytotoxicity. On the other hand, primary amines condense DNA better than other amines due to their higher protonation. Generally, L-PEI is a more efficient gene transfer agent than B-PEI due to its topology and lower cytotoxicity proved by in vivo studies despite its lower complexation capability toward nucleotides. B-PEI has the advantage of containing primary amino groups that are easily accessible to chemical modifications [19]. It should be noted, however, that the multiple reactive sites (i.e., primary and/or secondary amine groups) of PEI often cause complex coupling reactions that increase difficulties in obtaining compounds with controlled physical, chemical, and morphological characteristics, which are known to have an immediate impact on the TE [10,20]. One important focus during the last years was to develop approaches to considerably reduce the toxicity of the utilized PEI [21,22].

A convenient strategy to increase transfection efficiency with simultaneous decrease in cytotoxicity was found to be coupling of a low molecular weight PEIs (maximum 2000 Da) to a core connector forming conjugates of 8-54 kDa PEI [23,24,25].

In addition to the PEI toxicity subjects, the shielding of polyplexes charge by hydrophilic polymers, in particular polyethylene glycols, human serum albumin and dextran also called “stealth technology”, is another issue that has been intensively studied [25,26,27,28,29,30,31]. It has been shown that PEGylation of PEIs leads to an increased solubility of the complexes as well as to a reduction in the surfaces charge of the polyplexes [11,20]. Although PEGylation provided shielding and stealth property to PEI/DNA polyplexes, and, in some cases, they are also known to reduce the nonspecific ionic interactions between the complex and target cells, thus decreasing TE [7,23,32,33].

In the present study, we have addressed and investigated the above mentioned issues by applying a dynamic combinatorial chemistry approach developed by our group [34,35,36] to produce a series of new systems (dynamic combinatorial frameworks (DCFs)) containing PEGylated squalene (SQ-PEG-NH_2_) [34,37], poly-(ethyleneglycol)-bis(3-aminopropyl) (Mn~1500 g/mol) (NH_2_-PEG-HN_2_), and branched polyethylenimine of 2000 Da (PEI2000) components, reversibly connected in a hyperbranched structure. The obtained frameworks were capable to self-assemble in supramolecular amphiphilic architectures, driven by self-assembly properties of squalene derivatives, thus cumulating the number of PEI units per carrier. The prepared DCFs are capable of interaction with nucleic acids, forming stable polyplexes, and delivery of plasmid DNA to cells. The main focus of the study was to investigate the association between SQ-PEG-NH_2_, NH_2_-PEG-NH_2_ and PEI2000 in DCFs, for a deeper understanding of the structure-performance relation (in terms of transfection efficiency and tolerance in human cells) of such designed frameworks.

## 2. Results and Discussions

### 2.1. Formation of DCFs

Following the protocols developed in our group [34,38], a library of seven compounds (F1–F7, Table 1) was designed and prepared, containing 1,3,5-benzenetrialdehyde (TA) that serves as trifunctional core which reversibly connected functional moieties: (i) SQ-PEG-NH_2_ [34,37]; (ii) NH_2_-PEG-NH_2_; and (iii) PEI2000.

SQ-PEG-NH_2_ was utilized in the proposed design due to squalene derivatives biocompatibility [37,39,40] and, most importantly, due to its self-assembly ability in aqueous solutions [37,39,40,41]. The NH_2_-PEG-NH_2_ unit was chosen to shield the surface charge of the particles and to serve as a flexible linker for connecting components in frameworks via reversible imine bonds. Different amounts of PEI2000 units served as low molecular weight cationic binding sites that are able to efficiently bind DNA. The ratio of PEI2000 in the investigated DCFs was gradually modified from 1.5 equiv. to 3.5 equiv. in the composition of F1–F5, a slight excess of PEI was needed to establish the limit of toxicity. For the comparison results, F6 and F7 DCFs included 1 equiv. of NH_2_-PEG-NH_2_ in their composition. The formation of DCFs occurred in two steps; first, the reaction between TA and SQ-PEG-NH_2_ (or TA, SQ-PEG-NH_2_ and NH_2_-PEG-NH_2_) was performed in established ratio in organic solvent (acetonitrile). Next, after evaporation of the solvent, the residue was suspended in water followed by the addition of PEI2000 in established ratios according to Table 1.

**Figure 1:**
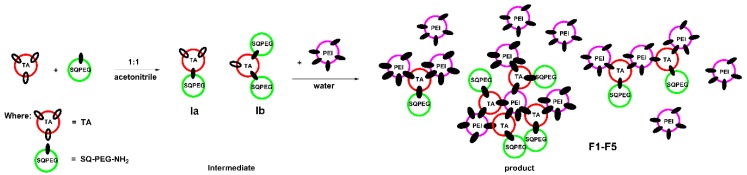
Schematic representation of the two-step synthesis of DCFs F1–F5.

**Figure 2:**
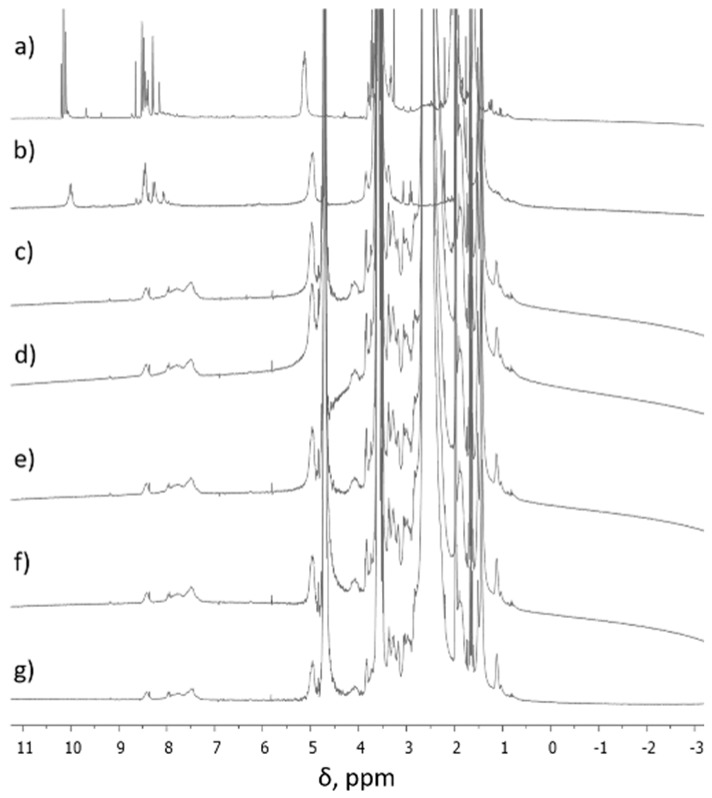
^1^H-NMR spectra of (**a**) intermediates (Ia, Ib) in CD_3_CN, (**b**) intermediates (Ia, Ib) in D_2_O, (**c**) F1 in D_2_O, (**d**) F2 in D_2_O, (**e**) F3 in D_2_O, (**f**) F4 in D_2_O, and (**g**) F5 in D_2_O.

During the formation of F1-F5 DCFs (Figure 1), the process was monitored by ^1^H-NMR, when the ratio between TA and SQ-PEG-NH_2_ in the first step was 1:1, and, as a consequence, the appearance of intermediates Ia and Ib was clearly observed (Figure 2).

Figure 2a,b shows ^1^H-NMR spectra (in different solvents) of the first step synthesis when TA interacted with SQ-PEG-NH_2_ in the 1:1 molar ratio and it was observed that the signal assigned to aldehydes (CH=O) at ~10 ppm was still present, indicating the existence of free carbonyl moieties. A deep introspection on signals at ~10 ppm shows the existence of three different carbonyl moieties (δ = 10.19; 10.15; 10.10 ppm), which were assigned to Ia, Ib, and unreacted carbonyl groups. Subsequent reaction of the intermediate mixture with the established amounts of PEI2000 resulted in total consumption of aldehyde moieties as can be seen in the ^1^H-NMR spectra, recorded in D_2_O (Figure 2c–g) and, as a result, the corresponding DCFs are formed.

**Figure 3:**
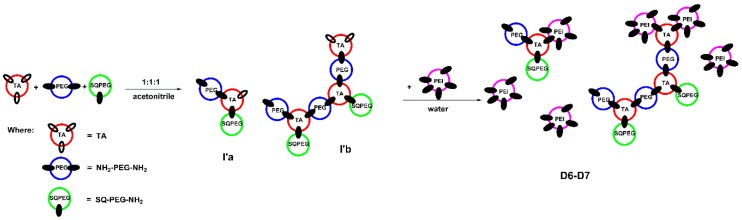
Schematic representation of the two-step synthesis of DCFs F6 and F7.

In case of DCFs F6 and F7, the results are similar to earlier described system [34]. As depicted in Figure 3, in the first step, when the ratio of TA:SQ-PEG-NH_2_:NH_2_-PEG-NH_2_ was 1:1:1, TA interacted with SQ-PEG-NH_2_ and NH_2_-PEG-NH_2_, leading to the formation of the mono and di-aldehyde intermediates (I’a and I’b, Figure 3), remarks supported by analyzing the ^1^H-NMR signals of the TA carbonyl moiety (Figure S1). Subsequent addition of NH_2_-PEG-NH_2_ to the composition of DCFs lead to the formation of branched structures (D6–D7, Figure 3). In the following step, when the intermediates were treated with various amounts of PEI2000, the ^1^H-NMR spectra showed the total consumption of aldehyde groups (Figure S1).

Additionally, after analyzing the ^1^H-NMR signals (Figure 2 and Figure S1), the aromatic and the imine proton signals of the investigated DCFs F1–F7 were highly broadened due to the formation of colloidal species, in which the squalene-driven process of self-assembling plays an important role in stability of the framework by its protection effect against the hydrolysis of the imine bonds [34].

### 2.2. Supramolecular Self-Assembling of DCFs

**Figure 4:**
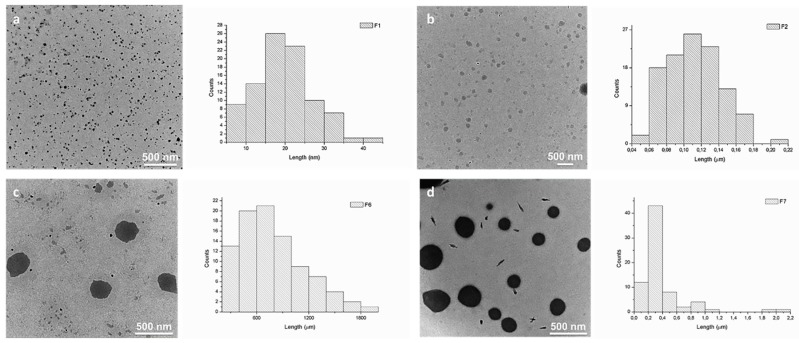
TEM images and corresponding average size distributions of the synthesized DCFs in water: (**a**) F1 (~30 nm); (**b**) F2 (~100 nm); (**c**) F6 (~500 nm); and (**d**) F7 (~300 nm).

We and others have earlier demonstrated that squalene derivatives are prone to self-assemble in aqueous media, forming amphiphilic compounds [37,39,40,41,42]. The self-assembly process of DCFs F1–F7 was studied and evaluated by TEM (Figure 4 and Figure S2). Analyzing TEM images, strong divergences in the size of DCFs containing NH_2_-PEG-NH_2_ (F6 and F7) when compared to DCFs lacking NH_2_-PEG-NH_2_ (F1–F5) were observed. Generally, the resulted F1–F5 amphiphilic DCFs containing SQ-PEG-NH_2_/TA/PEI2000 (Table 1), displayed uniform and small distinct spherical assemblies in water. Contrarily, in F6 and F7, major differences in the self-assembly processes were taking place; the recorded morphologies suggested the existence of large spherical morphologies (with diameters between 400 and 900 nm) with relatively narrow particle size distribution (Figure 4). These differences were most probably caused by the presence of the NH_2_-PEG-NH_2_ moiety in the DCFs composition, leading to a more complex cross-linking and subsequent self-assembling of the DCFs in larger particles.

### 2.3. AFM Studies

The AFM investigations of the DCFs clearly support TEM data (Figure S3), also showing a significant difference in the particle sizes of investigated DCFs allowing us to draw a clear conclusion that the lacking of NH_2_-PEG-NH_2_ in their composition caused the formation of much smaller spherical morphologies (F1 and F2) compared to F6 and F7. On the other hand, we could observe discrepancies between particle sizes of F6 and F7 determined by AFM (Figure S3c,d) and TEM (Figure 4c,d). Thus, in TEM, the size of F6 is slightly higher than that of F7, while AFM measurements show the opposite effect. Also, the size of the particles in TEM is higher (500 nm and 300 nm) than sizes determined by AFM (140 nm and 150 nm). The observed variances could be explained by the differences in nature of analyzed surfaces. The mica surface utilized for AFM investigations is negatively charged, thus favoring a stronger attachment of positively charged DCFs as is to the surface thus avoiding agglomeration of DCFs during the sample preparation. In the case of neutral carbon surfaces utilized in TEM grids, the analyzed DCFs may suffer partial agglomeration during drying out process, forming slightly larger particles. Similar effects in AFM and TEM analyses were observed earlier by Ursu et al. [43] during the investigation of charged entities.

Despite the observed size differences among same DCFs, both applied morphology methods (TEM and AFM) showed similar tendency of self-assembling process of DCFs in terms of size and shape of formed particles. Based on morphological studies, we assume that the difference in sizes occur due to dissimilar DCFs formation in two case scenario, while NH_2_-PEG-NH_2_ was or was not present in DCFs. The presence of NH_2_-PEG-NH_2_, being a linear polymer, will trigger the formation of larger DCFs, while, in the absence of NH_2_-PEG-NH_2_, PEI2000, which is a branched polymer, will lead to the formation of smaller branched DCFs.

### 2.4. DNA Binding

**Figure 5:**
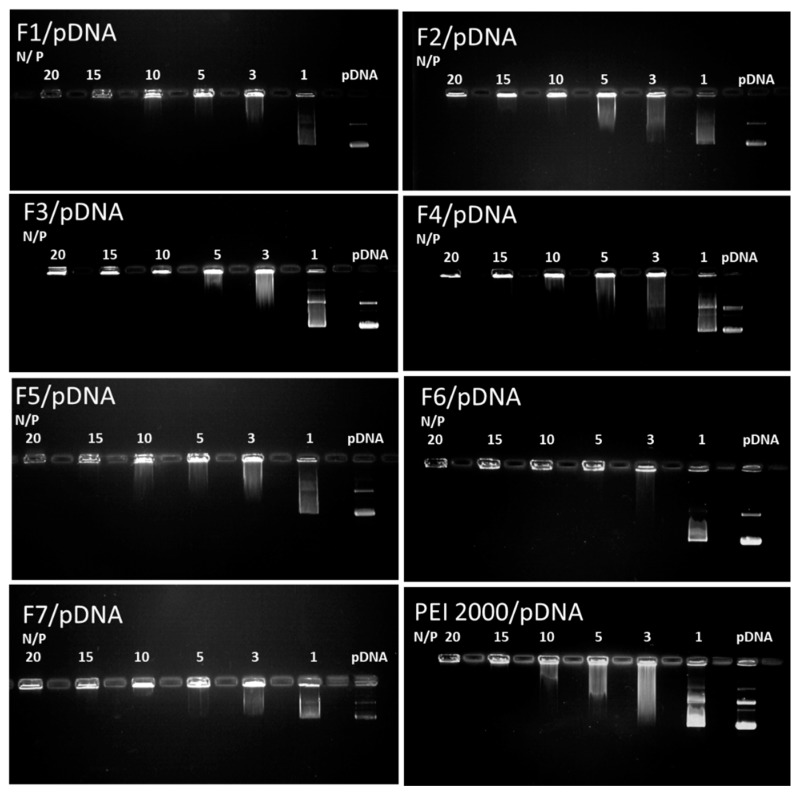
Images of agarose gel electrophoresis assays for F1–F7 compared to the reference PEI2000. Lane pDNA: naked DNA. Lanes 1–20: polyplexes F1-F7/pDNA, with the corresponding N/P ratios.

For gene delivery, the complexation ability of the carrier with DNA and the vector/DNA ratio are critical design parameters to control both the transfection efficiency and the cytotoxicity. The interaction abilities of F1–F7 with pDNA for cellular uptake were studied using gel electrophoresis reported to the reference parent PEI2000 (Figure 5).

Analysis of the results of gel retardation assays revealed the fact that all DCFs F1–F7 showed enhanced interaction with the investigated DNA. Interestingly, all DCFs were able to completely complex DNA at an N/P ratio of up to 10, whereas a higher N/P ratio of 15 was needed for the PEI2000 as reference.

### 2.5. DNA Binding Property by Gel Red Assay

An alternative method, the Gel Red (GR) dye exclusion assay, was utilized to confirm the gel retardation assay data in terms of the DNA binding ability of F1, F2, F6, and F7, and to gain more details on the DCF/DNA binding mechanism. In this method, GR, being sensitive, stable, and an environmentally safe fluorescent nucleic acid dye, was designed to replace the ethidium bromide for DNA staining [44]. The fluorescence of the GR significantly increases when dye molecules intercalate into double-stranded DNA, thus highest fluorescence signal is detected when the vector is absent or DNA is not interacting with the vector. Contrarily, the fluorescence signal becomes weaker when DNA binds to cationic polymer chains, sterically constraining dye–DNA interactions. The results of the GR exclusion assays have shown that F2 and F7 interacted much more strongly with DNA due to higher ratio of PEI than the F1 and F6 (Figure S4).

Starting with N/P ratio of 10, all analyzed DCFs possess enhanced DNA binding ability that no longer depended on the N/P ratio, data that confirmed the results from the Gel retardation assay. On the other hand, the GR dye exclusion assay did not distinguish differences in binding abilities below N/P ratio of 15, only suggesting that PEGylation of DCFs does not influence binding process of DNA, with the complexation process being strongly dependent on the PEI ratio in composition of DCFs.

### 2.6. Cytotoxicity and Transfection Efficiency

The cytotoxicity and TE of DCFs F1–F7 with pDNA (F1/pDNA-F7/pDNA) was investigated on HeLa cells by MTS assay, while in vitro TE was evaluated in HeLa cells using pCS2+MT-Luc DNA as a reporter gene. The cytotoxic evaluation showed that the toxicity of DCFs/DNA polyplexes in HeLa cells increased with the growing of PEI2000 ratio in composition of DCF. Thus, for F5/pDNA, the viability of HeLa cells was lower than 50% at the highest PEI content (3.5 equiv.). This cytotoxicity increase could be explained by the elevated number of primary amines of PEI. It was earlier shown that increased number of primary amines on the polymer, together with the surface charge, considerably reduce hemocompatibility and increase cytotoxicity displayed by PEI. [45] 

**Figure 6:**
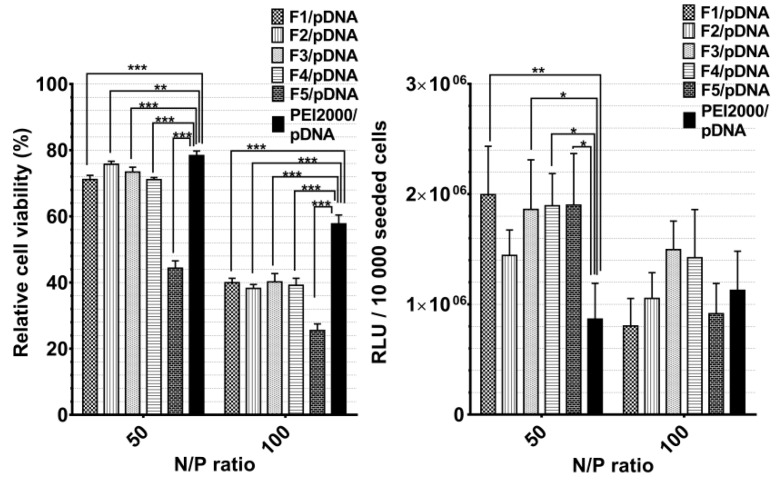
Graphical representation of relative viability and transfection efficiency (relative light units (RLUs)/10,000 seeded cells.) of Hela cells treated with polyplexes at 50 and 100 N/P ratios. The results are presented as a mean value ± the standard deviation (S.D.); n = 5–7. * *p* < 0.05, ** *p* < 0.01, and *** *p* < 0.001 by Student’s *t*-test.

Overall, when analyzing the cytotoxicity and the TE for the F1/pDNA-F7/pDNA, the following dependency was observed: with the increase of N/P ratio to 100, the cytotoxicity increased and the TE decreased (Figure 6).

Generally, the transfection efficiency depends on the vector structural characteristics and on its concentration related to the transported nucleic acids (N/P ratio). Notably, F1/pDNA-F5/pDNA exhibited markedly higher TE compared with PEI2000 at a N/P ratio of 50, regardless of the PEI2000 ratio in their composition. There was an insignificant difference in TE between F1/pDNA -F5/pDNA.

**Figure 7:**
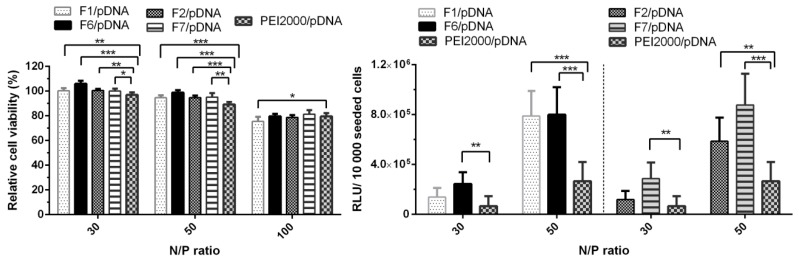
Graphical representation of relative viability and transfection efficiency (relative light units (RLUs)/10,000 seeded cells) of Hela cells treated with polyplexes at 30, 50 and/or 100 N/P ratios. The results are presented as a mean value ± the standard deviation (S.D.), n = 5–7. * *p* < 0.05, ** *p* < 0.01, and *** *p* < 0.001 by Student’s *t*-test.

Contrarily, TE considerably decreased with the increase of N/P ratio to 100. The obtained results suggested that optimal ratio of PEI in composition of DCFs is 1.5 equiv. and the most efficient N/P ratio is 50. Thus, the comparative cytotoxicity and transfection experiments for F6/pDNA and F7/pDNA were performed taking into account the optimal conditions. Next, PEGylated DCFs from F6/pDNA and F7/pDNA polyplexes were compared with non-PEGylated DCFs from F1/pDNA and F2/pDNA in order to evaluate the influence of NH_2_-PEG-NH_2_ on TE and cytotoxicity (Figure 7). PEGylation is frequently required for biocompatibility, steric stabilization due to its charge neutrality and water solubility, yet it is often associated with a decrease in TE [11,20,23].

As shown in Figure 7, all four analyzed polyplexes showed no obvious cytotoxicity at N/P ratios 30 and 50, as the viability of the cells treated with each of these polyplexes remained over 90%. A decreasing effect was observed at the highest N/P ratio of 100, the cell viability values dropped to 75–85%. Since all DCFs presented a lower TE at N/P ratio of 100 (Figure 6), only the N/P ratios of 30 and 50 were chosen for evaluation. Analyzing Figure 7, we could observe that the increase of N/P ratio from 30 to 50 led to an increase in TE, thus F1/pDNA, F2/pDNA, F6/pDNA, and F7/pDNA exhibited an improved transfection in comparison to the parent PEI2000/pDNA. Also, the presence of NH_2_-PEG-NH_2_ did not lead to the decrease in TE, on the contrary, it had a beneficiary influence in F7/pDNA complex.

## 3. Materials and Methods

### 3.1. Materials

Squalene (SQ) (purchased from Sigma, ≥98%), 1,3,5-benzenetrialdehyde (TA) (purchased from Manchester Organics, 98%), poly-(ethyleneglycol)-bis(3-aminopropyl) terminated (NH_2_-PEG-NH_2_) (Mn~1500 g/mol) (purchased from Aldrich, Slovakia), and branched polyethylenimine (2000 Da, 50 wt % in H_2_O) (PEI2000) (purchased from Aldrich, St. Louis, MO, USA). All other chemicals were purchased from Sigma-Aldrich Chemie GmbH (Steinheim, Germany) and used without further purification. PEGylated squalene (SQ-PEG-NH_2_) was prepared as previously described [34,37]. All reagents and solvents were purchased from commercial sources.

### 3.2. Experimental

#### 3.2.1. Synthesis of F1

In the first step, TA (1.3 mg) and SQ-PEG-NH_2_ (14.96 mg) were dissolved in 1000 µL acetonitrile. Reaction mixture was stirred for two days at room temperature. In the second step, the solvent was evaporated and residue was redissolved in 500 µL water and then PEI2000 (24 mg) dissolved in 396 µL water was added, the mixture was stirred for another 48 h at room temperature. Solution was kept as stock and used for further experiments.

#### 3.2.2. Synthesis of F2-F5

F2-F5 DCFs were synthesized according to the same protocol, changing the PEI2000 quantity in order to obtain a range of equiv. that vary from 2 (F2) to 3.5 (F5).

#### 3.2.3. Synthesis of F6 and F7

F6 and F7 were synthetized following the protocol described for F1 with the addition of 1 equiv. (12 mg) NH_2_-PEG-NH_2_ in the first step of the reaction. The quantity of PEI2000 was also varied to obtain the proposed composition with 1.5 equiv. of PEI2000 (F6) and 2 equiv. of PEI2000 (F7).

#### 3.2.4. Preparation of Polyplexes DCF/pDNA

The polyplexes with the plasmid DNA (pCS2+MT-Luc) from Harvard University, Boston were prepared at different molar ratios (N/P), considering the content of nitrogen from PEI in vectors, and the content of phosphate groups of plasmid DNA. Plasmid DNA (500 ng/µL) was mixed with the appropriate amounts of PEI and vector solution at N/P ratios of 30, 50, and 100, and incubated at room temperature for 30 to 60 min to generate vector/DNA polyplexes.

### 3.3. Methods

#### 3.3.1. Nuclear Magnetic Rezonance (NMR)

NMR spectra were recorded in CD_3_CN or D_2_O using a Bruker Avance III 400 instrument operated at 400.1 and 100.6 MHz for ^1^H and ^13^C nuclei respectively, at 25 °C. Chemical shifts (δ, ppm) have been described in relation to tetramethylsilane and coupling constants (J) are expressed in Hz.

#### 3.3.2. Transmission Electron Microscopy (TEM)

TEM images were obtained on a HT7700 Hitachi Transmission Electron Microscope. A volume of 3 µL of samples (F1–7) containing a concentration of 1.6 mg/mL PEI2000 Da were deposited on carbon coated copper grid and air dried for 24 h at ambient temperature. After drying, the samples were examined in high-resolution mode under an operative voltage of 100 kV.

#### 3.3.3. Atomic Force Microscopy (AFM)

The Ntegra Spectra Atomic Force Microscope (NT-MDT, Russia) operated in tapping mode under ambient conditions was used to image the polyplexes. Silicon cantilever tips (NSG 10, NT-MDT) with gold reflecting coating, a resonance frequency of 140–390 kHz, a force constant of 3.1–37.6 N m^−1^, and a tip curvature radius of 10 nm were used. Sample preparation: a 10-µL aliquot of the polyplex solution was deposited on freshly cleaved mica substrates and dried in air at room temperature prior to imaging.

#### 3.3.4. Agarose Gel Retardation Assay

An agarose gel retardation assay was applied to electrophoretically evaluate the formation of the polyplexes. Both the naked pCS2 plasmid DNA (0.5 μg) and the obtained polyplexes prepared from pCS2 plasmid DNA (0.5 μg) and F1-F7 at different N/P ratios (1, 3, 5, 10, 15, 20) were mixed with loading buffer (10× TAE buffer, pH 7.4), and then loaded per well in a 1% agarose gel. Electrophoresis was carried out at 90 V, for 90 min, in 1× TAE running buffer solution (40 mM Tris-HCl, 1% glacial acetic acid, 1 mM EDTA). The migration of free and complexed pCS2 was visualized and photographed under UV light, using a MiniBIS Pro system from (DNR Bio-Imaging), after staining with ethidium bromide (15 μL of 1% ethidium bromide in 300 mL double distilled water) and incubated for 20 min in dark at ambient conditions.

#### 3.3.5. Gel Red Exclusion Assay

The DNA condensation ability of vectors was determined by performing Gel Red exclusion assay. During the experiment, an aliquot of 10× Gel Red solution was prepared by diluting 10,000× Gel Red (Biotium) in ultrapure water. The diluted Gel Red solution (4 µL) was then added to naked pCS2 plasmid DNA solution (10 µL with 10 ng/µL of DNA, 0.1 μg) or each polyplex formed from F1–F7 and pCS2 plasmid DNA (0.1 µg) at N/P ratios of 2.5, 5, 7.5, 10, 15, 20, 35, 30, 40, and 50. The resultant mixtures were incubated at ambient temperature in the dark for 30 min. The fluorescence intensity of the mixture was measured using a plate reader (EnSight, PerkinElmer, Singapore) at excitation and emission wavelengths of 510 and 590 nm, respectively. The vectors were tested at N/P ratios between 0 and 50, where N/P 0 (DNA and GelRed) was considered 100% fluorescence intensity.

#### 3.3.6. Cell Cultures

HeLa cells (from CLS-Cell-Lines-Services-GmbH, Eppelheim, Germany) were cultivated in tissue culture flasks with alpha-MEM medium (Lonza) supplemented with 10% fetal bovine serum (FBS, Biochrom GmbH, Germany) and 1% penicillin–streptomycin–amphotericin B mixture (10K/10K/25 µg in 100 mL, Lonza). Medium was changed with fresh once every 3 or 4 days. Once confluency was reached, cells were washed with phosphate buffered saline (PBS, Invitrogen), detached with 1× Trypsin–Versene (EDTA) mixture (Lonza), centrifuged at 200× g for 3 min, and subcultured into new tissue culture flasks. All cell culture experiments were conducted under the same conditions: in the humidified incubator at 37 °C and 5% CO_2_.

#### 3.3.7. In Vitro Cytotoxicity Study (MTS Assay)

Cytotoxicity was measured using the CellTiter 96^®^ Aqueous One Solution Cell Proliferation Assay (Promega). HeLa cells were seeded into a 96-well culture plate at a density of 1 × 10^4^ cells per well in 100 µL culture medium (MEM medium supplemented with 10% fetal bovine serum (FBS) and 1% penicillin–streptomycin–amphotericin B mixture (10K/10K/25 µg)). After 24 h the medium in each well was replaced with 100 µL mixture containing fresh medium and polyplexes. Solutions of free PEI and F1–F7 were prepared as polyplexes, containing a final amount of 0.5 μg pCS2 plasmid DNA per well, at concentrations corresponding to N/P of 30, 50, and 100. After 44 h, 20 μL of CellTiter 96^®^ Aqueous One Solution reagent were added to each well, and the plates were incubated for another 4 h before reading the result. Absorbance at 490 nm was recorded with a plate reader (EnSight, PerkinElmer). Cell viability was calculated and expressed as percentage relative to viability of untreated cells. At least three biological replicates were used in the analysis. The results were represented as mean value ± standard deviation.

#### 3.3.8. In Vitro Gene Transfection Study

Twenty-four hours before transfection 1 × 10^4^ HeLa cells/well were seeded in a 96-well culture plate in a total volume of 100 µL/well. The loaded polyplex per well was obtained by mixing 1 µL plasmid (pCS2+MT-Luc) (500ng/µL) with a specific amount of F1–F7 solution that corresponds to the N/P ratio 30, 50, and 100. The mixture was incubated for one h at room temperature, to allow the formation of the polyplexes, and the culture medium (alpha-MEM medium supplemented with 10% fetal bovine serum (FBS) and 1% penicillin–streptomycin–amphotericin B mixture (10K/10K/25 µg)) was added, up to a total volume of 100 µL. The existing medium from each plate well was aspirated and the mixture of medium and polyplexes was added. At this point the cell monolayer was covered by this solution. After 48 h in the humidified incubator at 37 °C and 5% CO_2_, 100 µL Bright Glo Assay Reagent was added to each well, and the luminescence was measured within a 4 min interval. At least three biological replicates were used in the analysis. The results were represented as mean value ± standard deviation.

#### 3.3.9. Statistical Analysis

GraphPad Prism 6.04 for Windows (GraphPad Software, La Jolla California, CA, USA) was used to analyze data. At least 3 replicates were included in analysis. Results are presented as means ± standard deviation (S.D.). A Student’s *t*-test was used to compare two groups and the difference was considered significant when *p* < 0.05.

## 4. Conclusions

In order to understand the influence of the PEI ratio in the DCF composition on the crucial factors as self-assembling properties, DNA binding affinity, cytotoxicity and TE, a library of DCFs (F1–F7) was designed and synthesized based on 1,3,5-benzenetrialdehyde, PEGylated squalene derivative, poly-(ethyleneglycol)-bis(3-aminopropyl) terminated, and branched PEI of 2000 Da. Two DCFs were PEGylated, aiming to highlight differences caused by the presence of the NH_2_-PEG-NH_2_ moiety in the DCFs composition. Morphological studies (TEM and AFM) showed strong divergences in the size of DCFs containing NH_2_-PEG-NH_2_ (F6, F7) when compared to DCFs lacking NH_2_-PEG-NH_2_ (F1-F5), caused by complex cross-linking and the subsequent self-assembling of the DCFs in larger particles driven by NH_2_-PEG-NH_2_. DNA binding properties of DCFs were directly dependent on PEI ratio at low N/P ratios as presented in GR assays showing a stronger interaction with DNA when ratio of PEI is higher. The DCFs/DNA polyplexes, studied at N/P 50, became cytotoxic when the ratio of PEI in composition of DCFs reached 3.5 equiv. Increasing the N/P ratio to 100 led to increased cytotoxicity in HeLa cells, and the presence of NH_2_-PEG-NH_2_ did not induce significant improvement in cell viability. Overall, the studied DCFs exhibited distinctly higher TE compared with starting PEI2000 at N/P ratio of 50, regardless of the ratio of PEI in DCF composition and their TE considerably decreases with the increase of N/P ratio to 100. NH_2_-PEG-NH_2_ induces a subtle difference, improving the TE at N/P ratio 30 and 50. Thus, the obtained results suggested that optimal ratio of PEI in the composition of DCFs in this particular composition-wise scenario is 1.5 equiv., the most efficient N/P ratio is 50, and the presence of 1 equiv. of NH_2_-PEG-NH_2_ in order to achieve the best efficiency in transfection of pDNA to HeLa cells.

Overall, the investigated DCF approach in changing and testing the components for the improved transfection effect of DCF nonviral vectors, open a vast possibility in optimizing the structure of DCF by easy exchange of components. For example, we intend to also investigate the incorporation and testing of DCFs with PEG moieties of different sizes, exchange of linear and branched PEI of different sizes and last, but not least, the nature of the core molecule.

## Figures and Tables

**Table 1:** Composition (in equivalents) of the synthetized dynamic combinatorial frameworks (DCFs) library.

Compound	SQ-PEG-NH_2_	TA	NH_2_-PEG-NH_2_	PEI2000
**F1**	1	1	0	1.5
**F2**	1	1	0	2
**F3**	1	1	0	2.5
**F4**	1	1	0	3
**F5**	1	1	0	3.5
**F6**	1	1	1	1.5
**F7**	1	1	1	2

## Supplementary Materials

The following are available online, Figures S1–S4.
