# Supplementary material for: Synergistic Effect of Low Molecular Weight Polyethylenimine and Polyethylene Glycol Components in Dynamic Nonviral Vector Structure, Toxicity, and Transfection Efficiency
Source: Molecules. 2019 Apr 12;24(8):1460. doi: 10.3390/molecules24081460 (PMC6515267; doi:10.3390/molecules24081460)
Supplement: Supplementary file 1 [file molecules-24-01460-s001.pdf]

Synergistic effect of low molecular weight polyethylenimine and polyethylene glycol components in dynamic non-viral vectors structure, toxicity and transfection efficiency

Bogdan Florin Craciun, Gabriela Gavril, Dragos Peptanariu, Laura Elena Ursu, Lilia Clima, Mariana Pinteala

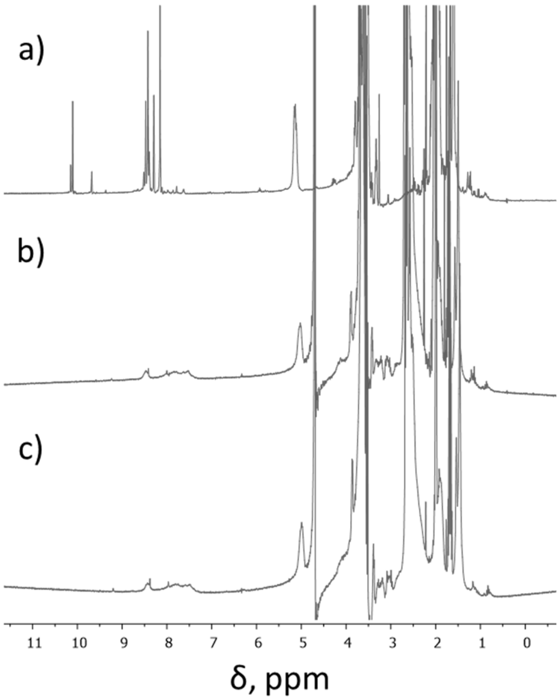

Figure S1. ^1H-NMR spectra of: a) Ia′, Ib′ intermediates in CD_3CN; b) F6 in D_2O, c) F7 in D_2O.

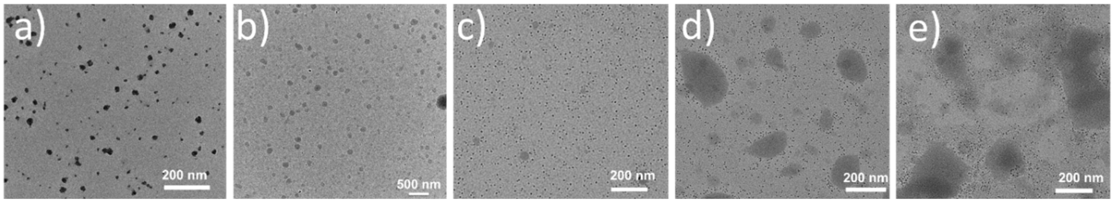

Figure S2. TEM images for the DCFs aqueous solutions: a) F1; b) F2; c) F3; d) F4; e) F5.

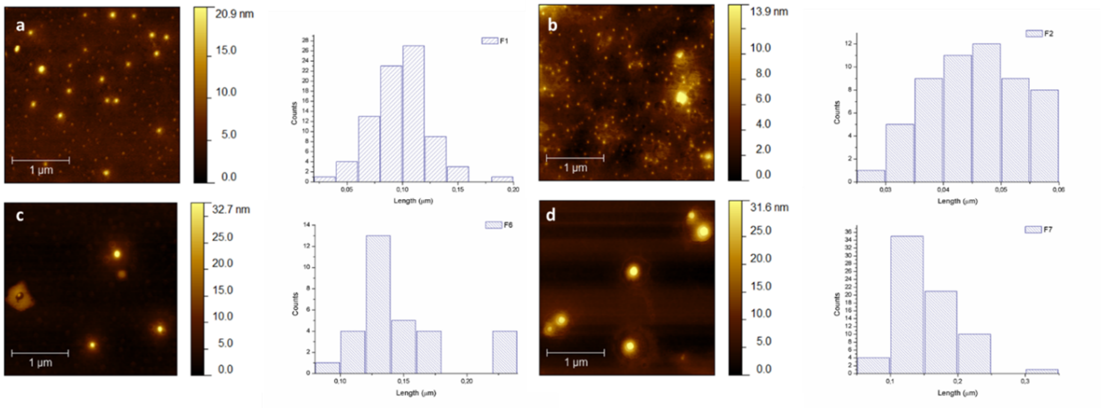

Figure S3. AFM images and corresponding average size distribution of the synthesized DCFs in water: a) F1 (~ 100 nm); b) F2 (~ 45 nm); c) F6 (~ 140 nm); d) F7 (~ 150 nm).

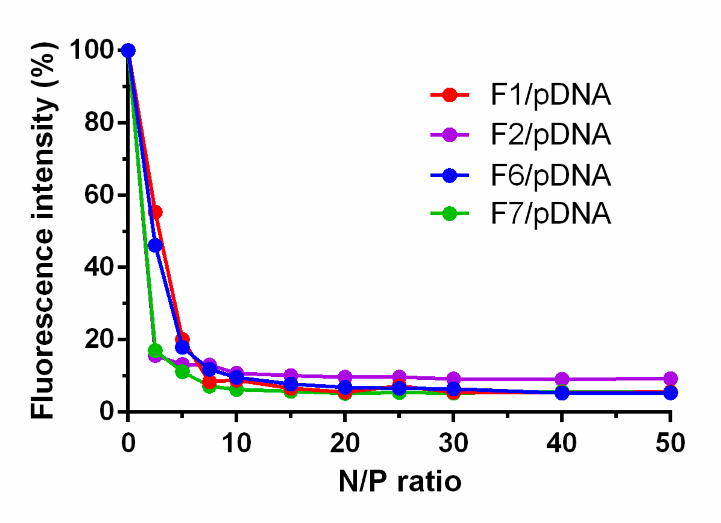

Figure S4. Gel Red dye exclusion assay. Fluorescence intensity of the Gel Red as a function of N/P ratio of formed polyplexes.
